# A Subovulatory Dose of Human Chorionic Gonadotropin (hCG) May Sustain Terminal Follicle Development and Reproductive Efficiency during Anestrus in Sheep

**DOI:** 10.3390/ani14071096

**Published:** 2024-04-04

**Authors:** José Francisco Cox, Albert Carrasco, Felipe Navarrete, Antonio Bocic, Fernando Saravia, Jesús Dorado

**Affiliations:** 1 Department of Animal Science, Faculty of Veterinary Sciences, Universidad de Concepción, Vicente Méndez 595, Chillán 3780000, Chileabocic@udec.cl (A.B.); fsaravia@udec.cl (F.S.); 2 Department of Medicine and Animal Surgery, Faculty of Veterinary Medicine, University of Cordoba, Campus Rabanales, 14014 Córdoba, Spain

**Keywords:** hCG, sheep, anestrus, estrous synchronization

## Abstract

**Simple Summary:**

Low reproductive performance during anestrus restricts the potential for year-round sheep production in temperate areas, precisely when food security is urgently demanded. Hormonal strategies used to improve this performance have largely relied on progesterone and equine chorionic gonadotropin (eCG). The last is a hormone obtained from the blood of pregnant mares that promotes follicular development (FSH activity in ruminants). However, recent information assigns LH, instead of FSH, the essential role of driving this development to ovulation. Therefore, we assessed this possibility using human chorionic gonadotropin (hCG) in anestrous sheep. The last has specific LH activity and is obtained from gestating woman urine. In this study, we showed that the fundamental restriction for better reproductive output during anestrus is a limitation not in the number of follicles but in their functional competence for ovulation. We also showed that a single administration of hCG seems as effective as eCG in promoting follicular development, ovulation, and fertility in sheep, provided that the hCG dose is appropriate. Our results could lead to better reproduction output during anestrus in sheep.

**Abstract:**

The study tested the hypothesis that a single administration of hCG supports the LH-dependent phase of terminal follicular development in synchronized sheep during anestrus, using eCG as a functional reference. Using a clinical approach, four experiments were designed to achieve the following: (1) Identify the inhibitory influence of anestrus on reproduction efficiency; (2) Assess the potential of hCG to keep functional blood concentrations after a single dose; (3) Characterize the effect of different doses of hCG on reproductive functional markers; (4) To compare the ability of hCG to that of eCG to support follicular development and fertility based on the same markers. The results showed that anestrus seems to affect follicular and luteal function under LH dependency as FSH-dependent markers are not compromised; hCG maintains higher blood concentrations than controls for at least 48 h; hCG improves follicular development and ovulatory rates compared to controls and at standards comparable to a breeding season; and ewes treated with hCG exhibit similar performance to those treated with eCG. Our results conclude that hCG can be used to support follicular function during anestrus in sheep, aiming to perfect its regulation in assisted reproduction.

## 1. Introduction

The suboptimal reproductive performance observed during anestrus presents a significant challenge for programmed sheep production in temperate regions [1,2]. Both the ovulation rate and fertility contribute to the low lambing yield observed during anestrus in most breeds. The number of follicles able to ovulate is an expression of follicular recruitment into the ovulatory wave and the follicular competence for ovulation; both are driven by the concerted activity of gonadotropins in interaction with insulin and insulin-like growth factors [3]. Female fertility, on the other hand, appears to be influenced by a wider array of factors, with the oocyte’s competence for fertilization and subsequent embryo development occupying a pivotal role [4,5].

Follicular stocks at recruitment are mainly under FSH regulation in sheep [6,7]. However, it remains ambiguous whether FSH serves as a fundamental constraint on ovulation rate during anestrus, given that the photoperiod does not appear to directly suppress the secretion of FSH from the pituitary gland in sheep [7,8]. Most probably, any influence of the photoperiod on the number of recruited follicles could be mediated by the ability of estradiol and inhibin A to shape FSH plasma concentrations during anestrus [9].

The acquisition of functional competence for steroidogenesis, ovulation, and fertility is the result of sound terminal follicular development, which appears to take place in the final 2–3 days leading up to ovulation [5,10]. Although FSH seems essential to trigger the ovulatory follicular wave, the terminal follicular differentiation that supports this functional competence is mainly under LH regulation in ruminants [11,12]. Indeed, any disruption to the frequency of LH pulses may potentially impair follicular function and fertility in sheep [11].

The inhibitory photoperiod reduces LH secretion from the pituitary in sheep. Indeed, there is consensus that extended photoperiods, via their ability to regulate the secretion patterns of melatonin from the pineal gland, expose KNDy neurons to the suppressive effect of dopaminergic neurons from the A15 nucleus, a process which is driven by follicular estradiol [13,14]. As a result, the length of the photoperiod can regulate the extent of the inhibitory influence of estradiol on the GnRH/LH pulse frequency and the outcome of terminal follicular development in sheep.

Existing protocols for estrous synchronization in sheep during the anestrous season [15] seem to overlook the complementary actions of both gonadotropins in driving terminal follicular development. In fact, equine chorionic gonadotropin (eCG) is used conventionally to support terminal follicular development. Although eCG can indeed support follicular growth and steroidogenesis [16] and narrow the interval to ovulation compared to untreated controls in ruminants [17], because of its FSH activity and its long biological activity that can even stimulate unscheduled follicular development, eCG has the potential to disrupt the endocrine balance supporting follicular functional differentiation [5,18] and the ovulation rate as observed in superovulation protocols [19,20,21]. Moreover, eCG carries additional issues that could limit its future use in assisted reproduction in ruminants; these include its ability to raise antibody titles [22], its significant contribution to the cost structure of synchronization protocols, and the perception of its processing protocol as highly invasive [23,24].

Human chorionic gonadotropin (hCG) has been used to support terminal follicular development in small ruminants during anestrus with conflicting results [25,26,27,28]. hCG is a glycoprotein structurally related to LH and can share LH receptors and biological activity [29]. It has a larger and more glycosylated structure than LH that slows down its clearance, allowing plasma concentrations to exceed 30 h in goats [30]. Considering that terminal follicle development in Highlander sheep requires 36–48 h [31], and since the pattern of LH secretion does not play a critical role in supporting its biological activity [11], a single administration of hCG could be sufficient to sustain this effect. McNatty et al. [32] also showed that follicles around 3 mm in diameter are the first to consistently respond to hCG, with an increase in cAMP following its administration in anestrous sheep.

Based on the above information, we hypothesize that a single administration of hCG at the end of a progesterone treatment can support terminal follicular development and oocyte competence for ovulation and fertility in anestrous sheep. We further propose that this response is comparable to that exhibited by ewes treated with eCG during the same period.

Therefore, the primary objectives of this study were to assess the effect of a single hCG administration on terminal follicle development, ovulation, and fertility in ewes and to compare these responses to those displayed by ewes treated with eCG at the same moment. A secondary aim was to compare terminal follicle development, ovulation, and fertility during both the breeding and anestrous seasons within the same group of Highlander ewes. These ewes were also pre-treated with a short-term progesterone protocol and exposed to male activity during the follicular phase.

The results supports our hypothesis as hCG added during the follicular phase was able to support terminal follicular development and fertility during anestrus in sheep. Furthermore, these results were comparable to those exhibited by ewes during the breeding season and by ewes treated with eCG.

## 2. Materials and Methods

### 2.1. General Procedures

#### 2.1.1. Animals and General Management

This study used non-lactating and clinically sound Highlander ewes, 2–4 years old, and 6 rams (2 Suffolk down and 4 Highlander, 2–4 years old) of known libido and fertility, identified using ear tags. The study was carried out at the animal facilities of the Faculty of Veterinary Sciences, Universidad de Concepción (36° south latitude, 71° west longitude). The breeding season of the Highlander breed at the campus lasts between February and July [27]. Experimental animals were used to the general management and personnel and were maintained in collective pens that considered adequate space for resting and feeding, ventilation, dry bedding, and free access to drinking water. During the day, the animals were allowed access to a 4-ha paddock for grazing and exercise. Feeding was based on alfalfa hay, oat grains, commercial concentrate, and mineral salts to maintain a body condition score (BCS) of around 3.0. Moreover, the animals were subjected to a preventive health program for endemic diseases. Housing and animal procedures followed standards approved by the Ethics Committee of the Faculty of Veterinary Sciences of the University (CBE-09-2024).

#### 2.1.2. Estrous Synchronization, Estrous Detection, and Mating Program

Ewes were synchronized via the hygienic introduction of an intravaginal device of progesterone (0.35 g progesterone, CIDR^®^ Ovino, Cooprinsem, Osorno, Chile) for 6 days followed by the administration of cloprostenol DL (0.125 mg i.m., Ciclase^®^, Syntex, Buenos Aires, Argentine) at CIDR removal. At that moment, their tag number was painted on their flanks to facilitate their identification later. After CIDR removal, estrous detection was carried out three times a day (08:00–09:00, 12:00–15:00, and 18:00–20:00) via direct observation of mount acceptance by intact rams. Rams were introduced to treated ewes in a male-to-female ratio of 1:8–10, and mated ewes were removed during the estrous detection period to a different pen to facilitate sexual search interaction. To visualize mating activity, males were rotated for each period of estrous detection. The rams were treated with melatonin (54 mg s.c., Melovine^®^, Ceva, Barcelona, Spain) 40 days before the mating activity to facilitate their sexual performance during anestrus. When mating was performed for pregnancy, each ewe was mated by at least two males before removing her from the group during the estrous detection period; otherwise, ewes were treated with Ciclase for luteolysis 4–5 days after estrus. The beginning of estrus was registered as the medium point between the last mating rejection period and the first estrous behavior; therefore, the interval to estrus was defined as the time elapsed between CIDR removal and the beginning of estrus. In addition, the estrous presentation rate was measured as the percentage of ewes detected in estrus compared to those treated.

#### 2.1.3. Blood Sampling and Endocrine Measures

Blood samples (3 mL) were collected via jugular venipuncture into heparinized glass tubes and were cooled to 5–10 °C until plasma collection (less than 2 h). Plasma was obtained via centrifugation at 5 °C (1500× *g*, 20 min), and samples were labeled and stored at −20 °C until assayed. Progesterone concentrations were measured via solid phase RIA using a commercial kit (PROG-RIA-CT, DiaSource, Louvain la Neuve, Belgium) validated for ruminants. The inter- and intra-assay CV were 4.3% and 5.0%, respectively, and the limit of sensitivity was 0.05 ng/mL. The hCG in plasma was measured using an IRMA and also using a commercial kit (hCG+β-IRMA, DiaSource, Ottignies-Louvain-la-Neuve, Belgium) that was validated for ewes and goats [33]. The intra-assay CV was 3.2%, and the limit of sensitivity was 1.6 IU/mL.

#### 2.1.4. Follicular and Corpora Lutea (CLs) Measures and Functional Definitions

Gonadotropin-dependent follicular development was monitored via transrectal ovarian ultrasonography (US) using a 10 MHz linear array probe connected to a B-mode, real-time scanner (Honda 2010 Vet, Toyohashi, Japan). The transducer was fitted to a plastic rod that allowed transrectal manipulation of the probe. Images were viewed at a magnification of ×2.0 with constant gain and focal point settings. Images of antral follicles and CLs were videotaped, frozen, and measured in mm using internal calipers. The mean diameter and relative location of all follicles ≥ 3.0 mm diameter [28] and CLs were sketched on ovarian charts for later analyses. The total luteal area was defined as the mean of the effective area of all CLs present in each ewe ((diameter^2^/4) × π). In those CLs that exhibited a cavity, the cavity area was subtracted from their area.

An ovulatory-sized (large) follicle was defined as a follicle ≥ 4.0 mm (minimum size registered for an ovulated follicle in this flock during anestrus). Ovulation was defined as the disappearance/collapse of large follicles between two consecutive US analyses followed by the growth of CLs 5–7 days after in the same locations; the time of ovulation was measured as the medium point between these two observations, and the interval to ovulation was measured as the time elapsed between the end of progesterone treatment and the time of ovulation. The term ovulated ewes was defined as the percentage of ewes exhibiting ovulation compared with those treated, and ovulation efficiency was defined as the percentage of preovulatory-sized follicles that exhibited ovulation compared with the number of ovulatory-sized follicles present in each ewe. The pregnancy rate was defined as the percentage of ewes pregnant compared with those treated; lambing rate as the percentage of ewes lambed compared with those pregnant; and fecundity rate as the percentage of lambs born per treated ewes. The analyses were performed using a double-blind criterium; that is, neither the ultrasound evaluator nor the personnel handling the ewes had access to the information on ewe allocation into experimental groups.

### 2.2. Experiment Descriptions

#### 2.2.1. Experiment 1: Reproductive Performance in Progesterone-Based Estrous Synchronized Highlander Ewes during Two Contrasting Periods of an Annual Reproductive Cycle

This experiment was performed to characterize the reproductive performance of Highlander ewes at the research unit under similar general management and subjected to a similar estrous synchronization and mating protocol, during the breeding season (April–June) and the anestrous season (October–December). Ewes (n = 79) were treated with the short-term progesterone protocol described above to synchronize their follicular phases. At CIDR removal (day 0), ewes were subjected to a transrectal US to verify the number and diameter of follicles ≥ 3.0 mm. In addition, at that moment, rams were introduced to the groups for mating and to impose a socio-sexual stimulation tone on the group [34]. Two days later (day 2), and on day 6 after estrus, US evaluations were performed to assess the number and diameter of large follicles present at that moment and to assess the number and total area of CLs, respectively. Moreover, at day 6 after estrus, ewes were sampled to measure plasma progesterone concentrations. Reproductive performance was assessed in terms of follicular and luteal development, estrous presentation, and ovulation performance and in terms of reproductive efficiency and lambing performance.

#### 2.2.2. Experiment 2: Plasma Concentrations of hCG after a Single Administration in Sheep

The study was carried out during April. Ewes (n = 17) were allocated at random into 3 experimental groups: ewes in Group 1 received 150 IU hCG (hCG 150, n = 6), those in Group 2 received 250 IU (hCG 250, n = 5), and ewes in Group 3 remained untreated (Control, n = 6). Blood samples were obtained just before hCG administration (0 h) and later at 6, 12, 24, 36, and 48 h after hCG administration, considering that the interval to estrus is about 36–48 h after short-term progesterone protocols [17]. To suppress endogenous LH pulse secretion, all ewes were pre-treated with intravaginal progesterone (0.35 g progesterone, CIDR^®^) for 6 days plus estradiol cypionate (0.3 mg i.m., ECP^®^, Zoetis, Santiago, Chile) at day 3 since the start of the CIDR treatment. hCG (Chorulon^®^, Intervet, AN Boxmeer, The Netherlands) was administered i.m. 12 h after the ECP treatment.

#### 2.2.3. Experiment 3: Effect of hCG on Terminal Follicular Development, Estrus, and Ovulation in the Anestrous Season

**Figure 1:**
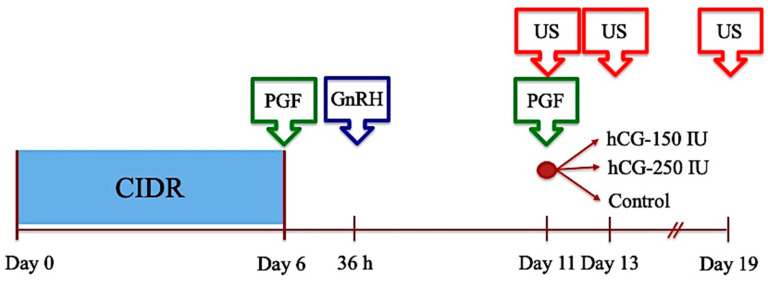
Experimental protocol to assess the effect of hCG on terminal follicular development under a synchronized follicular wave in sheep. PGF = cloprostenol; GnRH = buserelin acetate; US = ultrasound.

The study was carried out during September and January in two successive anestrous seasons and used 118 treated ewes distributed in 6 repetitions (Figure 1)—5 in the first season and an additional repetition in the second season (n = 18). To improve the dosage comparison, the hCG effect was assessed with ewes presenting a synchronized follicular dynamic at the start of the follicular phase. With that purpose, ewes were pre-synchronized using the 6-day CIDR protocol, followed by GnRH (Conceptal^®^, Intervet, Unterschieissheim, Germany) 36 h after CIDR removal. At day 5 after GnRH, ewes were treated with cloprostenol and were allocated randomly into 3 experimental groups: ewes in Group 1 received 150 IU hCG (hCG-150; n = 40), those in Group 2 received 250 IU hCG (hCG-250; n = 41), and ewes in Group 3 remained untreated (Control; n = 37). At that moment (day 0), ewes were subjected to a transrectal US to verify the presence of a CL and the number and diameter of follicles ≥ 3.0 mm. In addition, at that moment, rams were introduced to the groups to facilitate estrous detection and to impose a socio-sexual stimulation tone on the group [34]. Two days later (day 2), and on day 6 after estrus, US evaluations were performed to assess the number and diameter of large follicles present at that moment and to assess the number and total area of CLs, respectively. Other functional markers considered were estrous presentation, interval CIDR removal to estrus, and ovulated ewes. Three repetitions were used to assess the functional status of ovulations through markers of fertility (pregnancy rate, lambing rate, and fecundity rate). Therefore, the feeding and general management programs were adjusted to satisfy their requirements.

#### 2.2.4. Experiment 4: Comparative Assessment of the Effect of the Administration of hCG vs. eCG on Ovarian Functional Markers, Ovulation, and Fertility in Anestrous Sheep

The first part of the experiment was carried out at the University Campus between August and December and considered 80 ewes and 4 repetitions. Three repetitions were performed in one season and an extra one, which considered only a direct comparison between ewes treated with hCG and eCG, was performed in the following anestrous season. Ewes were considered as non-cycling by the absence of a CL after two US examinations separated by a week. Ewes were synchronized via the 6-day progesterone protocol used before. At the time of CIDR removal, ewes were allocated into 3 experimental groups: ewes in Group 1 received 150 IU hCG (hCG-150, n = 29), ewes in Group 2 received 150 IU hCG followed by an ovulatory dose of GnRH 36 h later (hCG+GnRH, n = 20), and ewes in Group 3 received 400 IU of eCG i.m. (Novormon^®^, Syntex, Buenos Aires, Argentine; eCG-400, n = 31). At that moment (day 0), ewes were marked on their flanks and rams were introduced to the experimental groups, mixed in collective pens, to facilitate estrous detection. US transrectal examinations were performed on days 0, 2, and 6 days after estrus to assess follicles and CL performances as before. To identify the moment of ovulation, US examinations were performed at 60, 72, and 84 h after CIDR removal to assess the evolution of preovulatory follicles. Blood samples were collected on days 5 and 8 after estrus to measure the plasma concentrations of progesterone (progesterone I and II, respectively). Therefore, in addition to follicular and CLs markers, an early increase in progesterone plasma concentrations, estrous presentation, interval CIDR–estrus, and interval CIDR–ovulation were also considered in this assessment.

In the second part of the experiment, the reproductive performance of Highlander sheep treated with hCG and eCG were compared directly in two commercial flocks managed similarly and with the same technical assistance, located in Osorno (40° south latitude, 73° west longitude) during December and February. The field experiment considered 112 Highlander ewes (n = 38 and n = 74 ewes in each farm) wearing ear tags, ≥3.0 BCS (1–5 scale), and 10 clinically sound rams (n = 4, 3–5-year-old rams and n = 6, 18-month-old ram hoggets, distributed in both flocks). Ewes were synchronized using the 6-day progesterone protocol used in this study; at CIDR removal, they were treated with cloprostenol and allocated at random into 3 experimental groups: ewes in Group 1 received 150 IU hCG (Chorulon-150, n = 39), ewes in Group 2 received 420 IU eCG (Novormon-420, n = 39), and those in Group 3 were left untreated (Control, n = 34). At that moment, the ram group, wearing harness markers, were introduced into the flock in a ram:ewe ratio of about 1:6 and were kept in the group. In farm 1, only one group of ewes was synchronized, whereas, in farm 2, two synchronization groups were considered; the second was synchronized using CIDR, as previously used, and rinsed and disinfected after their removal from the ewes from the first group [17]. Mating activity and lambing performance (ewes lambed after 147 ± 7 days after mating) were registered in both farms.

### 2.3. Statistical Analysis

Values are described as mean ± standard error of the mean (SEM). Most experiments were performed under similar animals, management, feeding and environmental influence. Thus, data were collected and analyzed to assess their distribution using the D’Agostino–Pearson normality test. In general, those that followed a normal distribution were compared by using either Student’s *t* test or a one-way analysis of variance (ANOVA) with a Tuckey post hoc test, when required, whereas those with nonparametric data were analyzed by using either the Mann–Whitney test or the Kruskal–Wallis test. Categorical data (estrous presentation, pregnancy, and lambing rates) were analyzed using either the Chi-Square test or Fisher’s exact test. Finally, hCG plasma profiles were analyzed using a two-way ANOVA for repeated measures. A *p* < 0.05 was considered significant. The Prism 10 (Graphpad Inc., La Jolla, CA, USA) statistical package was used for data management and analyses.

## 3. Results

### 3.1. Experiment 1: Characterization of Reproductive Performance of Highlander Ewes during an Annual Reproductive Cycle

This experiment was intended to assess the reproductive performance in ewes under the general management described above and subjected to the same estrous synchronization protocol as experimental ewes. The results are shown in Table 1.

The results in Table 1 show that Highlander ewes exhibit a consistent number of follicles entering terminal development, acquiring ovulatory size and ovulation competence, and also a similar number of post-ovulatory CLs (*p* > 0.10). However, depending on the annual season, they also exhibited differences in ovulatory follicular diameter, interval CIDR–estrus, total luteal area, and progesterone production, all functional markers that are associated to the production of estradiol and progesterone, which drive reproductive efficiency in sheep in terms of the fecundity rate (*p* < 0.05).

### 3.2. Experiment 2: Assessment of Plasma Concentrations of hCG in Ewes Treated with a Single Administration of 150 and 250 IU of hCG Compared to Untreated Controls

**Figure 2:**
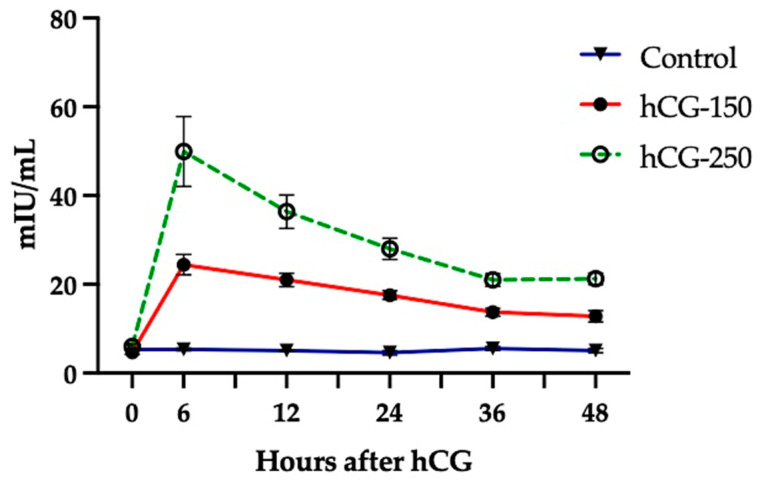
Plasma concentrations of hCG in ewes treated with a single administration of 150 and 250 IU hCG at time 0, compared to ewes left untreated as controls (mean ± SEM). Ewes were exposed to estradiol and progesterone to inhibit endogenous LH secretion.

Experimental ewes (n = 17) were subjected to the serial collection of blood during a 48 h period to describe their hCG profiles after treatment (Figure 2).

The results exhibit a significant influence of treatment, time, and the interaction between time and treatment (*p* < 0.0001). The single administration of hCG increased its plasma concentrations in a dose-dependent manner during the period considered, except at time 0 when all groups presented similar hCG plasma concentrations (*p* > 0.10). Untreated ewes exhibited a mean plasma concentration of 5.2 mIU/mL (%CV = 17.7) that was maintained during the collection period. Ewes treated with 150 IU hCG presented a mean plasma concentration of 17.9 mIU/mL (%CV = 18.8) and exhibited a peak 6 h after treatment in this sampling protocol, reaching plasma concentrations higher than those obtained at 36 and 48 h after treatment (24.5 vs. 13.7 and 12.8 mIU/mL, respectively; *p* < 0.01). Ewes treated with 250 IU hCG presented a mean plasma concentration of 31.3 mIU/mL (%CV = 21.9) that also exhibited a peak at 6 h after treatment reaching a mean plasma concentration of 50.0 mIU/mL that was higher than plasma concentrations in any other period (*p* < 0.001).

### 3.3. Experiment 3: Assessment of the Effect of the Administration of hCG on Terminal Follicular Development, Estrus, and Ovulation during the Anestrous Season

The study used 61 ewes and 118 treatments distributed in 6 replicates to gather information on ovarian response to hCG treatments. An analysis of fertility performance was carried out during December and January. No differences were found between replicates; therefore, data were grouped and compared directly. The results are shown in Table 2.

The results in Table 2 show similar numbers and diameters of follicles at CIDR removal; 48 h later, ewes treated with hCG had more large follicles and with larger diameters than untreated ewes (*p* < 0.05 and *p* < 0.005, respectively). Although ewes had similar estrous presentations, the interval from CIDR removal to estrus was shorter in ewes treated with 150 IU compared to untreated ewes (*p* = 0.005) and also with ewes treated with 250 IU (*p* = 0.04). hCG-treated ewes presented more CLs and mean luteal area than those ewes left untreated (*p* < 0.05 and *p* < 0.005, respectively); as a result of this different ovulatory rate, ewes treated with 150IU hCG presented a higher fecundity rate compared to untreated ewes (*p* < 0.01).

### 3.4. Experiment 4: Comparative Assessment of the Effect of the Administration of hCG vs. eCG on Ovarian Functional Markers, Ovulation, and Fertility in Anestrous Sheep

A total of 65 ewes and 80 treatments were used to assess this effect and the ability of GnRH to concentrate ovulations without affecting other functional markers of follicular function in ewes. The results are shown in Table 3.

The results in Table 3 show that functional markers considered in this study exhibited similar values except for the interval to ovulation where ewes treated with GnRH presented earlier and more concentrated ovulations compared to non-treated ewes (*p* = hCG: 0.004 and <0.001; eCG: 0.009 and 0.022 for the interval to and concentration of ovulations, respectively).

The reproductive efficiency in anestrous ewes synchronized by using the short-term progesterone protocol described above and treated with hCG and eCG were compared against untreated ewes under field management. A sub-group of ewes that had problems with their identification tags (n = 39) were removed from the experiment. The results are shown in Table 4.

The cumulative results in Table 4 show that ewes treated with hCG and eCG presented similar reproductive performance outcomes and a significantly superior performance in terms of mating activity (*p* < 0.0001), prolificacy rate (*p* = 0.002 and 0.007) and fecundity rate (*p* = 0.04 and 0.02 for hCG and eCG, respectively) than those exhibited by synchronized ewes left untreated as controls.

## 4. Discussion

The main findings of this study were that a single administration of hCG can maintain its plasma concentration above basal levels for at least 48 h. This is a sufficient period to support terminal follicular development up to ovulation programming in anestrous ewes. This ability is dependent on the hCG dosage and appears to be comparable to the ability of eCG to promote follicular development in anestrous sheep. Thus, these results support our hypothesis.

Additional information gathered in the study can also help to increase reproductive productivity during anestrus in sheep. As suggested, the number of FSH-dependent follicles at recruitment does not seem to be a fundamental constraint to the ovulatory rate during anestrous. Moreover, mating pressure could play a major role in increasing the fecundity rate during this period of the reproductive cycle.

Although there appears to be a lack of information on hCG blood concentrations after treatment in sheep, our results were expected due to the high degree of glycosylation shown by this glycoprotein (reviewed by [29]) and the pharmacokinetics displayed by hCG administered in goats [30]. The results show that blood concentrations of hCG remained above basal levels for 48 h, as in goats, and exhibited a similar bimodal curve characterized by an early peak followed by a gradual decrease in its plasma concentrations [30]. This period of activity is needed to support terminal follicle development, primarily under LH regulation [11,12,35], which ultimately leads to peak estradiol concentrations, the activation of estrous behavior, and the LH surge [36,37]. In addition, results show that even small changes in the hCG dosage (150 vs. 250 IU) produce significant differences in circulating hCG concentrations, with only minor variations between ewes throughout the sampling period.

As compared to untreated ewes, the administration of low doses of hCG appears to enhance follicular function during anestrus in sheep, as illustrated in Table 2. As expected, most functional markers affected by the treatments are consistent with the essential roles that LH has in sustaining follicular and oocyte competence for ovulation, luteal development, and fertility [3].

When compared to the reproductive performance of untreated but synchronized ewes in and out of the breeding season (Table 1), ewes from Experiment 3 exhibit similar features in both situations. The performance of hCG-treated ewes aligns with that of ewes during the breeding season, while the performance of control ewes aligns with the reproductive performance of ewes during anestrus. The number of follicles at the beginning of the follicular phase appears to be an exception to this general view. In Experiment 3, the count, but not the diameter, is lower in the hCG-250 group than in the other groups. However, the number of large follicles remained consistent throughout the study. This suggests that basal concentrations of FSH are enough to support follicular development up to ovulatory size [7,11]. Consequently, results also imply that the pattern of LH secretion is the primary limiting factor affecting sheep productivity during anestrus [38].

However, as mentioned above, there are conflicting results on the effect of using hCG during anestrus. Results in this study suggest that the effect of hCG depends on the dose used. Thus, as ewes treated with 150 IU in this study seem to exhibit better results collectively to those treated with 250 IU, it can be inferred that ewes treated with an even higher dose [26,27] would exhibit compromised terminal follicular development. LH is essential for terminal follicle development in sheep [11] and cows [12,35]; however, this final stage of development needs to be seen as a differentiation process in preparation for ovulation and luteal development—“follicle capacitation” [5,39]. This process seems to be based on a fine balance between proliferative driving forces, which are controlled by FSH, and differentiation signals, which are regulated by LH [5,40,41]. Therefore, it is valid to speculate that a strong LH signal may accelerate follicle differentiation and alter terminal follicle development, ovulation, and fertility.

The reproductive performance of ewes treated with hCG was evaluated in comparison to ewes treated with eCG. This comparison was made to assess the effectiveness of hCG against eCG, a long-standing gonadotropin conventionally used in assisted reproduction in small ruminants [23]. Interestingly, the ovarian and behavioral markers used in the eCG-treated group in this study mirrored the performance observed in an earlier study by Cox et al. on Suffolk ewes [17] and later in Highlander ewes [42]. Both studies used the same estrous synchronization protocol, similar doses of eCG, and comparable general management and feeding programs.

The results from Experiment 4 indicate that ewes treated with both gonadotropins (eCG and hCG) exhibited similar performance outcomes across all examined features. In addition, when the results in Experiment 4 were compared to the performance of untreated ewes shown in Table 1, eCG- and hCG-treated ewes perform similarly to ewes during their breeding season. This suggests that, during anestrus, both glycoproteins likely function by supplementing the sub-physiological concentrations of LH exhibited during this season [3,37]. Additional support for this hypothesis includes the pattern of follicle recruitment into follicular waves seen in this study and before [6,7], the lack of influence of the inhibitory photoperiod on FSH secretion [8], and the ligand specificity exhibited by the LH receptor [29].

The results in Experiment 4 also show that ewes treated with the GnRH agonist buserelin acetate, administered 36 h after CIDR removal, exhibited a significantly shorter and more concentrated interval to ovulation in hCG-treated ewes, in line with earlier results in eCG-treated ewes [43]. The interval to estrus, following CIDR removal, was about 30–36 h, as depicted in Table 3. Given that this interval depends on a peak secretion of estradiol, and this peak is also responsible for GnRH/LH surge activation [36,37], it suggests that the ovulatory response to GnRH was induced in a population of follicles already competent for ovulation. However, this follicle population was heterogeneous enough to exhibit less synchronized ovulation in the absence of GnRH, in line with the natural interval estrus–ovulation observed in small ruminants [44,45]. Therefore, GnRH treatment can be used for timed artificial insemination (TAI) year-round, as long as ewes are pre-treated with progesterone during anestrus [46].

The different fecundity rates observed in Experiment 1 and 3, compared to those in the field study in Experiment 4, have no clear explanation, even though such outcomes are commonly reported for synchronized ewes during anestrus [47,48]. The reproductive performance is an expression of multifactorial influence on the flock, as is the case with any species [1,47]. Given that factors such as the breed, health and feeding programs, and body condition score of experimental ewes did not appear to be limiting influences in this experiment, elements such as geographical location (40° vs. 36° south latitude) and ram activity could have potentially contributed to this outcome. We lack precise information regarding the duration of the breeding season in Osorno, as compared to our region; however, it is expected that the ability to resume ovulatory activity would vary depending on the geographic location of the flocks in relation to the Equator [38]. In addition, although we did not measure the rams’ influence on the reproductive outcome, the mating pressure imposed by the mating management in the university campus was higher than that used in the field study, as mating intensity requires infrastructure and ram management. The Ram Effect is a well-known, significant socio-sexual influence on mature ewes during anestrus [35]. It has been shown recently that, in addition to the libido and ram-to-ewe ratio, their time involved in sexual interaction may also play a significant influence on the reproductive outcome during anestrus [49]. Thus, the varying reproductive outcomes in this study could be viewed as a result of a combination of both hormonal and socio-sexual stimulation.

## 5. Conclusions

Cumulative information leads us to conclude that a single administration of a subovulatory dose (150 IU) can maintain hCG blood concentrations above basal levels for 48 h. This period is sufficient to sustain follicular functional competence that keeps ovulatory and fecundity rates at standards comparable to ewes during the breeding season. The reproductive response is similar to the one observed after eCG treatments. However, optimizing hCG dosage may still require information associated with the breed, geographical location of the flock, length of the photoperiod, and mating management. This is because it acts most probably by supplementing subphysiological concentrations of LH that are inhibited during the anestrous season in sheep.

## Figures and Tables

**Table 1:** Ovarian and reproductive performance (mean ± SEM) during an annual reproductive cycle of Highlander ewes synchronized by using a short-term progesterone protocol.

Parameters		Annual Reproductive Cycle		*p*-Value
		Breeding	Anestrus	
Number of ewes (n)		37	42	
Follicles ≥ 3.5 at day 0:				
	Number (n)	2.2 ± 0.19	2.3 ± 0.13	0.602
	Diameter (mm)	4.6 ± 0.09	4.4 ± 0.06	0.140
Follicles ≥ 4.0 at day 2:				
	Number (n)	2.4 ± 0.11	2.2 ± 0.15	0.175
	Diameter (mm)	5.5 ± 0.08	5.0 ± 0.08	<0.001
Estrous presentation (%)		100 (37/37)	88.1 (37/42)	0.057
Interval CIDR–estrus (h)		35.9 ± 1.62	41.1 ± 1.53	0.021
Ovulated ewes (%)		97.3 (36/37)	97.6 (41/42)	>0.999
Ovulating efficiency (%)		83.5 (71/85)	77.2 (71/92)	0.347
Corpora lutea at day 7:				
	Number (n)	1.9 ± 0.10	1.7 ± 0.10	0.076
	Total luteal area (mm)	173.0 ± 7.24	126.7 ± 7.72	<0.001
	Plasma progesterone (ng/mL)	6.5 ± 0.46	5.4 ± 0.24	0.018
Pregnancy rate (%)		97.3 (36/37)	76.2 (32/42)	0.008
Lambing rate (%)		88.9 (32/36)	96.9 (31/32)	0.361
Fecundity rate (%)		156.8 (58/37)	97.6 (41/42)	<0.001

**Table 2:** The effect of 2 doses of hCG (150 IU vs. 250 IU) on terminal follicle development (mean ± SEM), estrous presentation, ovulation, and fertility performance in Highlander ewes synchronized by using a short-term progesterone protocol and exposed to natural mating during the anestrous season.

Parameters		hCG-150	hCG-250	Control
Number of treatments (n)		40	41	37
Follicles ≥ 3.0 at day 0:				
	Number (n)	1.6 ± 0.18	1.3 ± 0.16	1.5 ± 0.19
	Diameter (mm)	4.6 ± 0.13	4.8 ± 0.11	4.5 ± 0.12
Follicles ≥ 4.0 at day 2:				
	Number (n)	2.5 ± 0.10 ^a1^	2.4 ± 0.15 ^a^	1.9 ± 0.12 ^b^
	Diameter (mm)	5.7 ± 0.09 ^a^	5.8 ± 0.10 ^a^	5.2 ± 0.10 ^b^
Estrous presentation (%)		97.5 (39/40)	87.8 (36/41)	91.9 (29/32)
Interval CIDR–estrus (h)		32.4 ± 1.50 ^a^	39.1 ± 2.41 ^b^	41.4 ± 1.91 ^b^
Ovulated ewes (%):		97.5 (39/40)	90.2 (37/41)	97.3 (36/37)
Corpora lutea at day 6:				
	Number (n)	2.3 ± 0.12 ^a^	2.0 ± 0.16 ^a^	1.5 ± 0.07 ^b^
	Total luteal area (mm)	157.3 ± 9.35 ^a^	143.3 ± 13.48 ^a^	92.8 ± 5.95 ^b^
Pregnancy rate (%):		94.7 (18/19)	76.2 (16/21)	89.5 (17/19)
Lambing rate (%):		89.5 (17/19)	71.4 (15/21)	84.2 (16/19)
Fecundity rate (n):		168.4 (32/19) ^a^	133.3 (28/21) ^ab^	115.8 (22/19) ^b^

^1^ Different superscripts in the same row indicate statistical differences (*p* < 0.05).

**Table 3:** Comparative performance of progesterone-synchronized ewes treated with hCG, hCG plus GnRH, and eCG on terminal follicle development (mean ± SEM), estrous presentation, and ovulation performance.

Variables		hCG-150	hCG-GnRH	eCG-400
Number of ewes (replicates)		29 (4)	20 (3)	31 (4)
Follicles ≥ 3.0 at day 0:				
	Number (n)	1.9 ± 0.20	2.3 ± 0.22	2.3 ± 0.23
	Diameter (mm)	4.7 ± 0.11	4.8 ± 0.13	4.8 ± 0.11
Follicles ≥ 4.0 at day 2:				
	Number (n)	2.3 ± 0.14	2.3 ± 0.19	2.5 ± 0.16
	Diameter (mm)	6.2 ± 0.14	6.0 ± 0.17	6.0 ± 0.12
Estrous presentation (%)		89.7 (26/29)	85.0 (17/20)	87.1 (27/31)
Interval CIDR–estrus (h)		32.6 ± 1.52	32.2 ± 1.36	33.1 ± 1.46
Ovulated ewes (%):		93.1 (27/29)	85.0 (17/20)	90.3 (28/31)
Interval CIDR–ovulation (h)		67.3 ± 2.45 ^a1^	55.9 ± 1.27 ^b^	65.8 ± 1.90 ^a^
Corpora lutea at day 6:				
	Number (n)	2.2 ± 0.16	1.7 ± 0.21	1.6 ± 0.16
	Total luteal area (mm)	166.0 ± 9.78	146.3 ± 14.23	153.7 ± 10.0
	Progesterone I (ng/mL) ^2^	3.5 ± 0.26	NA	4.1 ± 0.46
	Progesterone II (ng/mL)	4.8 ± 0.45	NA	6.9 ± 0.78

^1^ Different superscripts in the same row indicate statistical differences (*p* < 0.05); ^2^ plasma progesterone concentrations 7 and 10 days after CIDR removal. Statistical analyses showed differences in groups with different superscripts. The precise difference is in the text.

**Table 4:** Comparative responses in estrous presentation and fertility of progesterone-synchronized Highlander ewes treated with hCG and eCG.

Farm	Reproductive Parameters	Experimental Groups		
		hCG-150	eCG-420	Control
1	Mating activity (%)	11/13 (84.6)	11/13 (84.6)	5/12 (41.7)
	Lambing rate (%)	6/11 (54.5)	8/11 (72.7)	3/5 (60.0)
	Prolificacy rate (n)	13/6 (2.17)	16/8 (2.0)	3/3 (1.0)
	Fecundity rate (%)	13/13 (100)	16/13 (123.1)	3/12 (25.0)
2	Mating activity (%)	22/26 (84.6)	25/26 (96.2)	9/22 (40.9)
	Lambing rate (%)	12/22 (54.5)	12/25 (48.0)	6/9 (66.7)
	Prolificacy rate (n)	20/12 (1.67)	18/12 (1.5)	6/6 (1.0)
	Fecundity rate (%)	20/26 (76.9)	18/26 (69.2)	6/22 (27.3)
Total	Mating activity (%)	33/39 (84.6) ^a1^	36/39 (92.3) ^a^	10/34 (29.4) ^b^
	Lambing rate (%)	18/33 (54.5)	20/36 (55.5)	9/14 (64.3)
	Prolificacy rate (n)	33/18 (1.83) ^a^	34/20 (1.70) ^a^	9/9 (1.0) ^b^
	Fecundity rate (%)	33/39 (84.6) ^a^	34/39 (87.2) ^a^	9/34 (26.5) ^b^

^1^ Different superscripts in the same row indicate significant differences (*p* < 0.05). Statistical analyses showed differences in groups with different superscripts. The precise difference is in the text.

## Data Availability

The datasets presented in this article are not readily available because they are part of an ongoing study. Requests to access the datasets should be directed to J.F.C.
